# Dual Stimuli-Responsive P(NIPAAm-*co*-SPA) Copolymers: Synthesis and Response in Solution and in Films

**DOI:** 10.3390/polym10060645

**Published:** 2018-06-09

**Authors:** Oliver Grimm, Felix H. Schacher

**Affiliations:** 1 Institute of Organic Chemistry and Macromolecular Chemistry (IOMC), Friedrich-Schiller-University Jena, Humboldtstraße 10, D-07743 Jena, Germany; oliver.grimm@uni-jena.de; 2 Jena Center for Soft Matter (JCSM), Friedrich-Schiller-University Jena, Philosophenweg 7, D-07743 Jena, Germany

**Keywords:** dual-stimuli-responsive materials, spiropyran, NIPAAm, controlled radical polymerization

## Abstract

We present the synthesis and solution properties of dual stimuli-responsive poly(*N*-isopropylacrylamide-*co*-spiropyran acrylate) (P(NIPAAm-*co*-SPA)) copolymers of varying composition prepared *via* nitroxide-mediated copolymerization. The resulting copolymers feature molar masses from 40,000 to 100,000 g/mol according to static light scattering and an SPA content of up to 5.3%. The latter was determined by ^1^H NMR spectroscopy and UV–Vis spectroscopy. These materials exhibit reversible response upon irradiation in polymeric films for a minimum of three cycles; their response in solution to both light and temperature was also investigated in an aqueous TRIS buffer (pH 8). Irradiation was carried out using LED setups with wavelengths of 365 and 590 nm. In aqueous solution, a custom-made setup using a fiber-coupled 200 W Hg(Xe) lamp with 340 and 540 nm filters was used and additional heating of the copolymer solutions during irradiation allowed to study influence of the presence of either the spiropyran or merocyanine form on the cloud point temperature. Hereby, it was found that increasing the SPA content leads to a more pronounced difference between both states and decreasing cloud points in general.

## 1. Introduction

Over the last decades, numerous polymeric materials that respond to an external stimulus such as temperature, pH value, or ionic strength have been reported. Among these examples of stimuli-responsive materials, fewer studies have reported materials that can respond to more than one trigger, and, in the best case, two orthogonal stimuli have been reported. Such dual stimuli-responsive polymers are attracting greater attention for application in a vast number of fields from drug delivery and sensing to actuators [1,2,3,4,5,6]. Among the available triggers, temperature is the most applied and extensively investigated, with various studies exploiting the lower critical solution temperature (LCST) of different polymers. Prevalent examples of such polymers include poly(*N*-isopropylacrylamide) (PNIPAAm) [7,8], poly(2-oxazolines) [9,10,11,12], polypropylene glycol [13,14,15], or poly(oligo(ethylene glycol) acrylates) [16,17,18].

PNIPAAm exhibits a lower critical solution temperature (LCST) in water at 32 °C [19], and since its discovery in 1968 has served as a key example for temperature-responsive polymers in aqueous media [20]. Nevertheless, even after 50 years of research, a coherent quantitative picture remains elusive, as stated in a recent review by Halperlin [8]. Upon heating above the LCST, such materials undergo a coil-to-globule transition and, depending on further factors such as the overall concentration, precipitate from aqueous solution [7]. Therein, the formation of hydrogen bonds plays a significant role, and in most cases aqueous solutions are investigated [21]. The broadening of the chain length distribution also typically affects the transition temperature. Additionally, the incorporation of a comonomer or the presence of various salts can be used to tune the transition temperature of such materials [22].

**Scheme 1:**
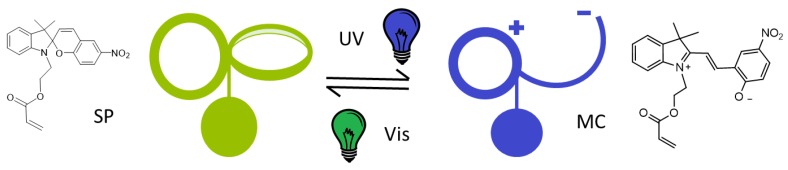
Photo-responsive switching between the spiropyran (SP) (**left**) and merocyanine (MC) forms (**right**) of a potential benzo-spiropyran monomer.

As another example, light-responsive materials can also react reversibly by, *e.g.*, isomerization, or irreversibly by photo-cleavage upon exposure to light of a certain wavelength [23]. The application of light as a stimulus is particularly interesting, as it is able to be applied with both temporal and spatial control. There are a broad range of examples of photo-responsive low-molecular-weight molecules. Fihey [24] and Bleger [25] identified the most prominent photo-responsive molecules: azobenzenes [26,27], diarylethenes [28,29,30,31], and spiropyrans. Numerous derivatives of these molecules have been synthesized to tune the excitation wavelength, response kinetics, and photostability, and to allow these functional moieties to be incorporated into polymeric materials. Spiropyrans were first synthesized by Löwenbein and Katz in 1926 [32]. In 1972, the first polymeric materials featuring spiropyran moieties were reported by Smets [33]. Depending on the substitution pattern, the photo-response of this class of molecules can be additionally tuned [34] and, based on these insights, a large variety of substitution patterns for polymerizable spiropyrans have been synthesized [35]. In the case of spiropyrans, a reversible switching from the closed, nonpolar spiropyran form to the planar, zwitterionic merocyanine form occurs upon irradiation with UV light (Scheme 1) [36,37,38]. Stimulation with visible light (~550 nm) then leads to ring closure and the spiropyran is again formed. This change in polarity renders such materials interesting for a large number of applications including surfaces featuring controlled wetting [39] or photo-responsive micellar nanostructures [40].

The combination of thermo-responsive PNIPAAm and photo-responsive PSPA is intriguing; depending on the spiropyran state, the polarity, charge density, and, thus, temperature-dependent solution properties of any copolymer can be controlled [41,42,43]. In that regard, the presence of the zwitterionic merocyanine form should concurrently increase the hydrophilicity and, therefore, the cloud point temperature [35]. This effect will also depend on the spiropyran content in the copolymer [44], or—as already shown—in hydrogels featuring swelling and de-swelling depending on irradiation with different wavelengths [45,46]. The free radical copolymerization of linear P(NIPAAm-*co*-SPA) was reported by Sumaru and coworkers in 2004 [42,47,48,49]. They investigated the effect of irradiation and varying pH on the temperature response of aqueous solutions. Later on, they used comparable copolymers in photo- and thermo-responsive membranes [50]. Such SPA comonomers have also been used in atom transfer radical polymerization by Achilleos and coworkers [51], and in grafting-from approaches towards the preparation of dual-stimuli-responsive SiO_2_ nanoparticles [52]. Moreover, the introduction of SPA-based monomers into block copolymers that undergo self-assembly in selective solvents to form polymeric micelles allows nanostructures to be created that respond to various stimuli [53]. Recent reports have also demonstrated that the micellar morphology can be switched from worm-like to spherical [54], and that such structures can carry and release cargo through the successful encapsulation and release of doxorubicin [55].

We herein report on the synthesis and characterisation of linear and dual-responsive poly(*N*-isopropylacrylamide-*co*-spiropyran acrylate) (P(NIPAAm-*co*-SPA) copolymers using nitroxide-mediated polymerization (NMP). The application of NMP here seems as a facile method to both allow control over the copolymer composition and the actual content of photo-responsive moieties as well as the possibility to prepare (amphiphilic) block copolymers using sequential polymerization steps. Different compositions were synthesized and characterized *via* size exclusion chromatography (SEC), static light scattering (SLS), and nuclear magnetic resonance spectroscopy (NMR). We were able to further show that the resulting copolymers are photo-responsive in the solid state using different irradiation wavelengths in polymeric films. Additionally, in aqueous media both the response to irradiation with light as well as to heating above the cloud point of the copolymers could be demonstrated using a custom-made setup. We were able to show that the cloud point depends not only on the overall content of SPA, but also on the switching state of the spiropyran moieties. The kinetics of the photo-response from both films and solutions were compared to the results of diluted solutions of SPA in organic media.

## 2. Materials and Methods

### 2.1. General Information

2-bromoethanol (95.0%), 2,3,3-trimethyl-3H-indol (97.0%), 2-hydroxy-5-nitrobenzaldehyde (97.0%), and *N*-isopropylacrylamide (98.0%) were purchased from TCI (Zwijndrecht, Belgium) and used as received. Acryloyl chloride (97.0%) was purchased from Sigma-Aldrich (Munich, Germany) and used as received. All solvents were of analytical grade except 1,4-dioxane and THF, which were purchased from Carl Roth (Karlsruhe, Germany) in HPLC grade.

NMR measurements were carried out on a 300 MHz Bruker NMR spectrometer (Karlsruhe, Germany). The solvent used is always specified, and the spectra were referenced to the solvent signal.

Size exclusion chromatography (SEC) was performed on a Shimadzu system (Kyoto, Japan) equipped with an SCL-10A VP system controller, an LC-10AD VP pump, an RID-10A refractive index detector, and an SPD-10AD VP UV detector at 365 nm using a solvent mixture containing Dimethylacetamide (DMAc) and 0.21% LiCl at a flow rate of 1 mL·min^−1^ on two PSS GRAM guard columns (1000 and 30 Å with a separation range of approx. 400–1,000,000 g/mol). The system was calibrated with polystyrene standards from PSS (Mainz, Germany).

For irradiation in the solid state, two LEDs at 365 nm (0.8 mW) and two LEDs with 590 nm (22 cd) from Roithner (Vienna, Austria) were placed in front of the sample, resulting in an estimated light intensity of 3.4 mW/cm^2^ for the 365 nm light source, and 4.5 mw/cm^2^ for the 590 nm LED in the setup used.

For irradiation in solution, a 200 W Hg(Xe) lamp from LOT-QuantumDesign (Darmstadt, Germany) was used. The light was filtered by a UV (U340 from Edmund Optics, Karlsruhe, Germany) and green filter (VG-9 from Edmund Optics) coupled into a glass fiber and placed over the sample. The estimated light intensity for the aqueous setup was 10.6 mW/cm^2^ for both wavelengths.

The UV–Vis measurements were performed on an Agilent (Santa Clara, CA, USA) Cary 60 UV–Vis spectrophotometer with a peltier single cell holder at the given temperatures. For the solid-state UV–Vis measurements, the solid samples were placed in a custom-made sample holder with the LEDs placed in front of them. The measurements in solution (for both irradiation and temperature dependence) were performed in a cuvette (Hellma Optics, Jena, Germany) with a path length of 1 cm. The irradiation source was in both cases placed in a custom-made holder to ensure the position remained the same for every measurement.

The static light scattering measurements were performed using an ALV Laser CGS3 Goniometer (Langen, Germany) equipped with an ALV Avalanche correlator and a He–Ne laser (λ = 633 nm). All SLS measurements were performed at 25 °C. To determine the molecular weight, three measurements of 30 sec each with a difference of less than 5% were performed at angles between 30° and 150° at a step width of 10°. The copolymers were dissolved in HPLC-grade THF at concentrations of 1, 2, 3, 5, and 10 g/L. The *q*^2^ + *k*c values were plotted in a zimm-plot against *K*_c_/*R* to determine the mass-averaged molecular weight. 

### 2.2. Synthesis of 1-(2-Hydroxyethyl)-2,3,3-trimethyl-3H-indolium Bromide

A mixture of 2,3,3-trimethyl-3H-indol (2.72 mL, 16 mmol) and 2-bromoethanol (1.48 mL, 20 mmol, 1.25 eq) in acetonitrile (20 mL) was purged with argon and heated for 26 h under reflux at 100 °C. The solvent was removed and resuspended in 25 mL hexane. The collected solid was recrystallized from chloroform and directly used in the following step.

### 2.3. Synthesis of 9,9,9a-Trimethyl-2,3,9,9a-tetrahydro-oxazolo[2,3-a]indole

1-(2-Hydroxyethyl)-2,3,3-trimethyl-3H-indolium bromide **1** was dissolved in water and mixed with KOH (0.66 g, 12 mmol). The mixture turned from pink to yellow within 10 min at room temperature. The mixture was then extracted with diethylether (3 × 20 mL), dried over MgSO_4_, filtered, and the solvent removed under reduced pressure. The yellow oil (0.8549 g, 33.5%) was analyzed *via* ^1^H NMR:

^1^H NMR (300 MHz, CDCl_3_): δ = 1.89 (s, 3H, CH_3_), 1.31 (s, 3H, CH_3_), 1.42 (s, 3H, CH_3_), 3.44–3.86 (m, 4H, 2 × CH_2_), 6.76 (d, 1H, CH-arom.), 6.93 (t, 1H, CH-arom.), 7.14 (m, 2H) ppm.

### 2.4. Synthesis of 2-(3′,3′-Dimethyl-6-nitro-3′H-spiro[chromene-2,2′-indol]-1′-yl)-ethanol

9,9,9a-trimethyl-2,3,9,9a-tetrahydro-oxazolo[2,3-*a*]indole **2** (0.85 g, 4,2 mmol, 1 eq) and 2-hydroxy-5-nitrobenzaldehyde (1.054 g, 6.3 mmol, 1.5 eq) were mixed in 10 mL of ethanol and heated under reflux for 3 h. After cooling to room temperature, the remaining solution was filtered and washed with ethanol. The red crystals (0.6319 g, 33%) were analyzed *via* ^1^H NMR:

^1^H NMR (300 MHz, DMSO-d_6_): δ = 1.10 (s, 3H, CH_3_), 1.20 (s, 3H, CH_3_), 3.19 (dq, 2H, CH_2_), 3.44 (m, 2H, CH_2_), 4.72 (t, 1H, OH), 6.01 (d, 1H, CH-arom.), 6.64 (d, 1H, CH-arom.), 6.78 (t, 1H, CH-arom.), 6.87 (d, 1H, CH-arom.), 7.11 (m, 3H, 3 × CH-arom.), 8.00 (dd, 1H, CH-arom.), 8.21 (d, 1H, CH-arom) ppm.

### 2.5. Synthesis of 2-(3′,3′-Dimethyl-6-nitrospiro[chromene-2,2′-indolin]-1′-yl)ethyl Acrylate (SPA)

2-(3′,3′-Dimethyl-6-nitro-3′H-spiro[chromene-2,2′-indol]-1′-yl)-ethanol **3** (0.5 g, 1.42 mmol) and trimethylamine (0.197 mL, 1.42 mmol) were dissolved in dichloromethane, purged with argon, and cooled to −35 °C. A solution of acryloyl chloride in dichloromethane was added slowly and the reaction mixture was heated up to room temperature overnight. The solution was extracted with saturated NaHCO_3_ (2 × 20 mL) and water (2 × 20 mL), and the organic phases were combined, dried over MgSO_4_, filtered, and evacuated under reduced pressure. The product was purified via column chromatography with chloroform to yield 107.6 mg (0.265 mmol, 18.6%).

^1^H NMR (300 MHz, DMSO-d_6_): δ = 1.06 (s, 3H, CH_3_), 1.19 (s, 3H, CH_3_), 3.36–3.51 (m, 2 × 1H, CH_2_), 4.16–4.35 (m, 2 × 1H, CH_2_), 5.90 (d, 1H, CH_2_) 5.96 (d, 1H, CH-arom.), 6.09 (dd, 1H, CH), 6.26 (d, 1H, CH_2_), 6.72 (d, 1H, CH-arom.), 6.80 (t, 1H, CH-arom.), 6.85 (d, 1H, CH-arom.), 7.12 (m, 2H, 2 × CH-arom.), 7.20 (d, 1H, CH-arom.), 7.99 (dd, 1H, CH-arom.), 8.21 (d, 1H, CH-arom.) ppm.

### 2.6. Synthesis of P(NIPAAm-co-SPA)

In a typical preparation, SPA (4 mol % SPA, 155 mg), *N*-isopropylacrylamide (1043 mg), and *1-tert*-butyl-3,3-dipropyl-5,5-diethyl-4-(1-phenylethoxy)-piperazin-2-one (10 mg NMP initiator M/I = 400) were dissolved in 3 mL dioxane in a microwave vial. The solution was purged with argon for 30 min and heated to 110 °C for 41 h. The resulting red solution was precipitated twice into cold diethylether, resulting in a red powder (600.6 mg, 49.7% yield). All mentioned compositions (0%, 1%, 2%, 3%, 5%, and 6%) were synthesized using this protocol. The copolymers were analyzed *via* SEC and ^1^H NMR in CDCl_3_.

^1^H NMR (300 MHz, CDCl_3_): δ = 4 (s, 1H, isopropyl), 6 (s, 1H, SPA), 6.8 (m, 3H, SPA), 8 (d, 1H, SPA arom.), 8.2 (s, 1H, SPA arom.) ppm.

DMAc-SEC: 0%: 22,900 g·mol^−1^ (2.47), 1%: 25,700 g·mol^−1^ (1.86), 2%: 25,800 g·mol^−1^ (1.76), 3%: 25,500 g·mol^−1^ (1.58), 4%: 26,300 g·mol^−1^ (1.55), 19,300 g·mol^−1^ (1.47), 6%: 22,600 g·mol^−1^ (1.41).

## 3. Results and Discussion

### 3.1. Synthesis of the Spiropyran Acrylate (SPA) Monomer

**Scheme 2:**
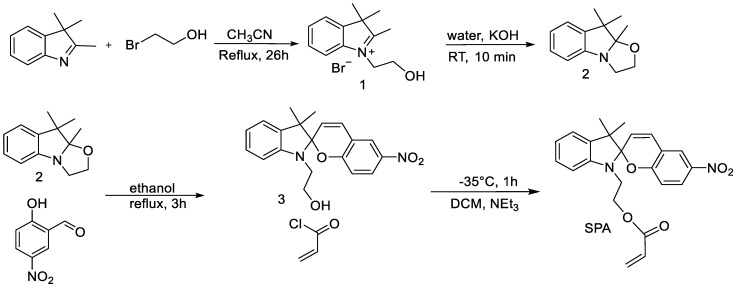
General procedure for the synthesis of the spiropyran acrylate (SPA).

**Figure 1:**
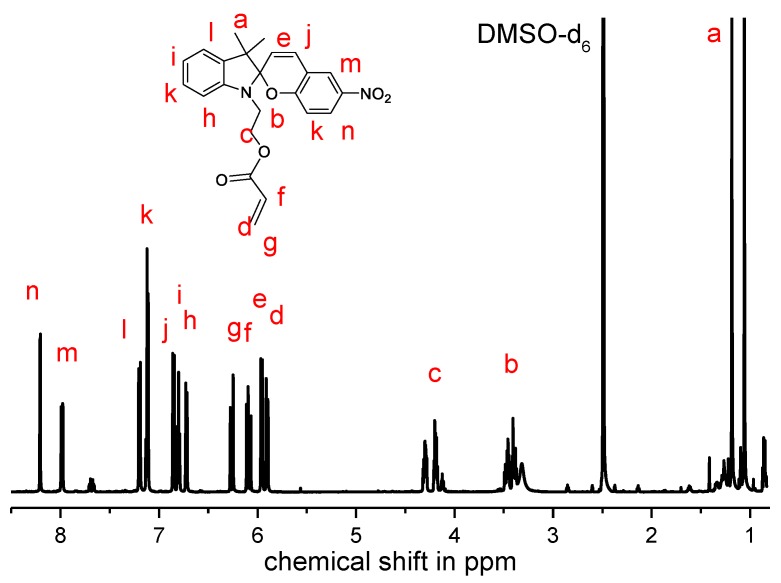
^1^H-NMR spectrum of SPA in DMSO-d_6_.

The polymerizable spiropyran derivative was synthesized according to a procedure reported by Raymo and Giordani [56] and Matyjaszewski et al. [57] and depicted in Scheme 2. Briefly, 2,3,3-trimethyl-3H-indol was mixed with 2-bromoethanol and heated to form **1**. Without further purification, **1** was dissolved in water at pH 14 to form **2**. The brown liquid was reacted with 2-hydroxy-5-nitrobenzaldehyd in ethanol under reflux to form **3**. Finally, the monomer SPA was formed as a blue oil at low temperatures, and turned into pale yellow crystals overnight, which were afterwards stored in the dark. The ^1^H-NMR of SPA shows all expected signals (Figure 1).

The C_2_-linker provides sufficient mobility, and the primary alcohol is a convenient reactive handle that can undergo numerous modifications. In addition, the substitution pattern of the spiro-group provides rapid photo-response rates, and attachment of the polymerizable group at the nitrogen has already been shown to render such monomers suitable for emulsion polymerizations [58].

### 3.2. Synthesis of P(NIPAAm-co-SPA) Copolymers

**Figure 2:**
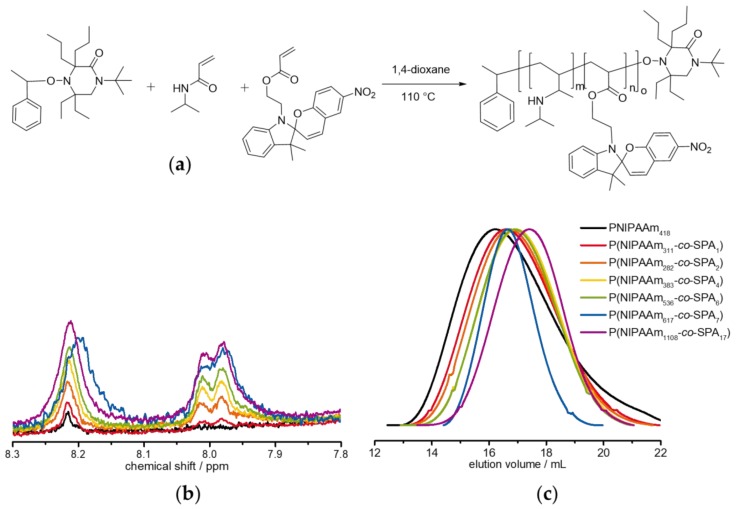
(**a**) Reaction scheme depicting the synthesis of poly(*N*-isopropylacrylamide-*co*-spiropyran acrylate) (P(NIPAAm-*co*-SPA)). (**b**) Detailed view of the ^1^H-NMR signals of different P(NIPAAm-*co*-SPA) in DMSO-d_6_; the signal at ~8 ppm was used to calculate the SPA content in the copolymers. (**c**) Normalized RI SEC elution traces of the copolymers using DMAc as eluent.

Dual responsive copolymers of varying composition were synthesized using nitroxide-mediated polymerization (NMP). NIPAAm and SPA (up to 6 mol % in the initial monomer mixture) were dissolved in 1,4-dioxane. 1-*Tert*-butyl-3,3-dipropyl-5,5-diethyl-4-(1-phenylethoxy)-piperazin-2-one was added as initiator, which had been synthesized according to literature protocols [59,60]. The reaction mixture was heated to 110 °C overnight, and during this time a color change from green to red was observed. The resulting copolymers were precipitated twice in diethylether to remove unreacted monomer and solvent, and the prepared copolymer was collected by filtration. The yield decreases with increasing SPA amount in the copolymerization mixture. The monomer conversion as determined by NMR decreases with increasing SPA content. After 8 h the conversion for the reaction mixture containing 5% SPA reaches 50%, whereas the reaction without SPA already shows full conversion according to NMR. We attribute this to steric hindrance of the SPA comonomer. The prepared copolymers were analyzed *via* size exclusion chromatography (SEC) with dimethylacetamide (DMAc) as eluent, ^1^H NMR in DMSO-d_6_, static light scattering (SLS), and UV–Vis spectroscopy (Figure 2 and Figure S1).

After the synthesis and purification of the prepared copolymers by multiple precipitations of seven different compositions, the ^1^H NMR spectra showed no remaining monomer signals (see Figure S1). By normalization of the corresponding spectra to the isopropyl proton of NIPAAm at 4 ppm, the integral at 8 ppm increases with the amount of incorporated SPA comonomer. The SEC elution traces show monomodal distributions and corresponding dispersities ranging from 1.41 to 2.47 with number-averaged molecular weights near 24,000 g/mol (Table 1). The absolute molar mass and copolymer composition were determined by static light scattering (SLS) in tetrahydrofuran (THF), which well solubilizes the prepared copolymers. In general, higher molar masses are obtained by SLS in comparison to SEC (PS calibration). However, the rather high dispersities of the samples as well as the formation of loose aggregates at higher SPA contents may further complicate the SLS measurements.

The amount of SPA in the copolymers was determined using two different approaches: ^1^H-NMR in CDCl_3_, and from the absorption coefficient of the copolymers in THF. For the determination via ^1^H-NMR, the signals at 4 (1H, NIPAAm) and 8 ppm (2H, SPA) were compared. As shown in Figure S1b, the signals of the C_2_-Linker from the SPA at near 4.2 ppm overlap with the isopropyl signal for NIPAAm at near 4 ppm in the copolymers, and were therefore subtracted to determine corrected SPA contents. As the signal-to-noise ratio from ^1^H-NMR is poor at low amounts of SPA, UV–Vis spectroscopy as an alternative method was applied to confirm the calculated compositions.

Comparison of the molar extinction coefficient of the monomer and the respective copolymers (Figure S2), based on the assumption that the molar extinction coefficient of the SPA comonomer is comparable to the SPA units in the copolymer, was used to determine the composition. As seen in Table 1, a similar trend as observed in the NMR experiments but higher SPA contents are determined using UV–Vis spectroscopy. This is more pronounced for P(NIPAAm_1108_-*co*-SPA_17_), where 5.5% (NMR) and 10.8% (UV–Vis) are found. As we cannot exclude the occurrence of π–π stacking at higher SPA fractions, it is our opinion that the SPA content is overestimated by UV–Vis. All synthesized copolymers were soluble in aqueous media at room temperature in the dark. As the copolymers with SPA contents exceeding 6% exhibited cloud point temperatures distinctly below room temperature, we refrained from using higher SPA fractions in the following experiments.

### 3.3. Photo-Response in the Solid State

**Figure 3:**
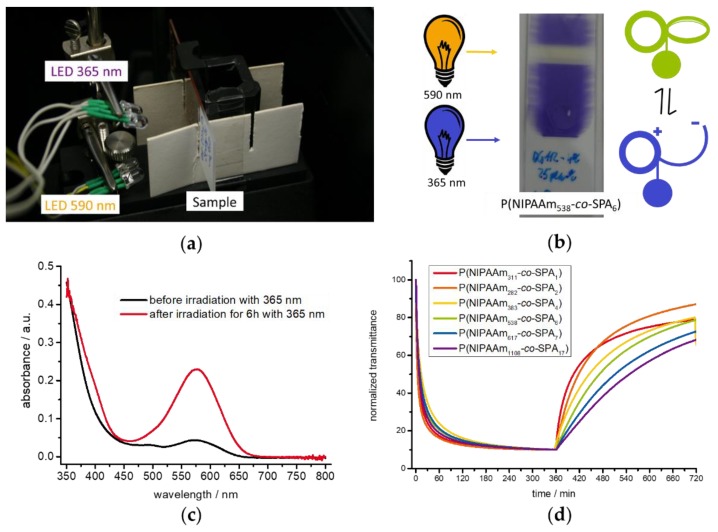
(**a**) Custom-made setup used to measure the transmittance of the copolymer films during irradiation at 365 or 590 nm; (**b**) P(NIPAAm_538_-*co*-SPA_6_) film on a glass slide after partial irradiation; (**c**) Absorption spectra of P(NIPAAm_282_-*co*-SPA_2_) in the solid state before irradiation at 365 nm, and after reaching equilibrium after 6 h; (**d**) Normalized transmittance after one switching cycle of P(NIPAAm-*co*-SPA) containing varying amounts of SPA. The absorption was monitored at 550 nm after irradiation at 365 and 590 nm for 6 h each.

The synthesized copolymers already display a visible color change in the solid state, switching from yellow to violet upon irradiation with UV light. To investigate this behavior, a solution of 25 wt % of the respective copolymer in THF was doctor bladed with 120 µm step height onto a glass substrate. The thickness of the resulting copolymer films was determined via profilometry and varied from 11 to 17 µm, presenting a moderately homogeneous surface (Figure S3). In a custom-made sample holder for the UV–Vis spectrometer, the films were irradiated with LEDs at either 365 or 590 nm while measuring the transmittance [61] at 550 nm over at least three cycles with an irradiation time of 6 h for every wavelength (Figure 3). Irradiation with an increased light intensity using a 200 W Hg(Xe) lamp using a 340 and 540 nm filter in a similar setup did not increase the reaction rate constants in the described copolymer films. Upon irradiation at 365 nm, the transmittance at 550 nm decreases, increasing again upon irradiation at 590 nm. During irradiation at 365 nm, the SPA units in the copolymer are switched from the closed spiropyran to the opened merocyanine form whereas irradiation at 590 nm reverses this process. This can be observed by the decreasing transmission at 550 nm during irradiation, and after 6 h the system reaches equilibrium. At this point, ring closure is induced by irradiation at 590 nm.

During the first 6 h, the transmittance of the glass slides decreases to 10%, and increases again during irradiation at 590 nm as the SPA is switched to the spiropyran form. The transmittance after the first cycle clearly shows that a higher transmittance is obtained for copolymers containing lower amounts of SPA. In addition, the films were switchable for a minimum of at least three cycles for each copolymer sample (Figure S4). However, cracks appear on the film surface during the irradiation cycles that can be attributed to mechanical stress [62]. Depending on the amount of SPA present in the respective copolymer, this is accompanied by a decrease in transmission due to crack formation. The reaction rate constants are comparable over the three cycles, and we also observed this effect when the switching was carried out within a sealed microscope slide under argon. Another general trend observed is that higher amounts of SPA in the copolymer result in increased absorption, and thus exhibit different kinetics in response to irradiation. To visualize this, the first switching cycle was normalized with respect to transmittance (Figure 3d). As can be seen, the photo-response of all copolymers is, in general, faster during irradiation with UV light. Single molecule experiments have shown that the photochemical conversion to the merocyanine form is up to ten times faster than the reverse photoreaction [63]. In our case, the re-formation of the spiropyran form occurs more slowly with increasing SPA content, as these units receive less photons per chromophore at an identical light intensity.

### 3.4. Photo-Response in Aqueous Solution

The above-discussed P(NIPAAm-*co*-SPA) copolymers can be expected to be both temperature and light-responsive in aqueous solution due to the cloud point temperature of PNIPAAm and the reversible photo-switching of PSPA [20,37]. Nevertheless, the response of spiropyran derivatives to irradiation with light depends on several factors, particularly in aqueous environment, including light [64], pH value [35], the presence of certain counterions [65], mechanical stress [66], and temperature as the ring closure is also promoted at higher temperatures [67].

First, investigations on the response of the copolymers in aqueous solution to irradiation were carried out to elucidate whether the process is reversible, and determine the limitations for external trigger such as, e.g., pH value. The optimal conditions for the irradiation response were then applied to determine differences in cloud point temperature depending on whether the respective sample is predominantly in the spiropyran or merocyanine form.

The nitrogen in the SPA moiety of the copolymer can be protonated. According to the literature [68], the p*K*_a_ for single molecules in the closed spiro form is approximately 0.5, while the phenolic hydroxyl group of the open merocyanine form has a p*K*_a_ of approximately 4. Furthermore, the merocyanine form can decompose upon hydrolysis [69] or by photodegradation [70,71]. All five switching states (two protonated, two unprotonated, and the decomposed SPA) have different UV–Vis absorption maxima, which are described by Hammarson et al. [68]. To compare the behavior reported for single molecules to that of the copolymers described here, solutions of P(NIPAAm_538_-*co*-SPA_6_) at different pH values were prepared. The samples were irradiated for 30 min with either 340 or 540 nm filtered light from a 200 W Hg(Xe) fiber-coupled light source. The Hg(Xe) light source provides a higher light intensity at the sample compared to the previously used LEDs, and, therefore, a faster response is obtained. The absorbance spectra after 30 min of irradiation were recorded (Figure S5), as well as the change in absorbance at 550 nm over time (Figure S6). Prior to irradiation, a mixture of the spiropyran and merocyanine forms is present due to thermal equilibration; however, this can be strongly influenced by irradiation [72]. The photo-response can be described by pseudo-first-order kinetics, and these are provided in the Supporting Information (Figures S7 and S8). In general, the same kinetic constants were observed for both the copolymers and single molecules. From this observation, we may assume that the p*K*_a_ values may also be comparable. If we consider a p*K*_a_ value of 4, then at pH 8 the phenolic hydroxyl group of the merocyanine form is approximately 93% deprotonated to assure an adequate switching behavior. Hence, all investigated copolymers were irradiated using a 200 W fiber-coupled Hg(Xe) lamp for 30 min with either a 340 or 540 nm filter for three consecutive cycles (see Figure S9). In general, the maximum absorbance decreases after every cycle for about 6% to 12%, which we attribute to (i) the protonation of approximately 7% of SPA molecules in the merocyanine form that are stabilized in this state, and (ii) some photodegradation processes that are occurring [70,71].

**Figure 4:**
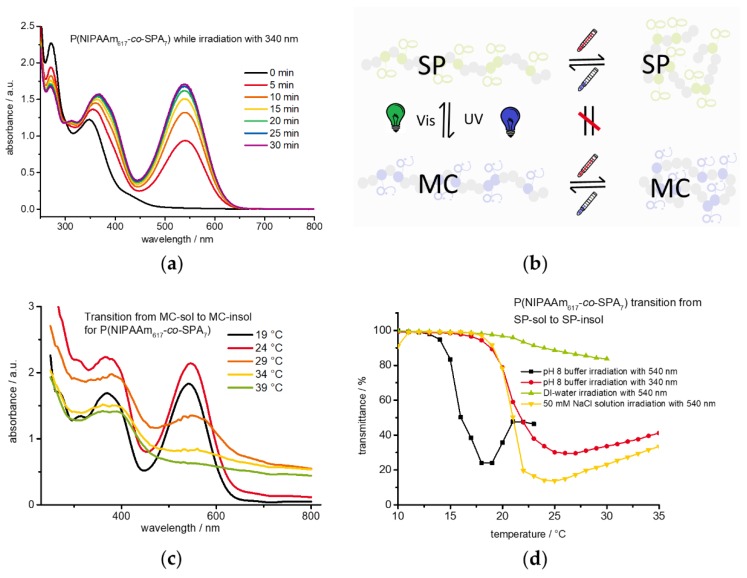
(**a**) UV–Vis spectra of P(NIPAAm_617_-*co*-SPA_7_) in a pH 8 buffer while being irradiated at 340 nm at 15 °C; (**b**) Switching scheme depicting the four different states adopted by the copolymers; (**c**) UV–Vis spectra of P(NIPAAm_617_-*co*-SPA_7_) in a pH 8 buffer while being irradiated at 340 nm at different temperatures; (**d**) Transmittance of P(NIPAAm_617_-*co*-SPA_7_) at 700 nm with increasing temperatures in various aqueous solutions.

For the investigation of the irradiation response of the copolymers, the respective copolymer was dissolved in a pH 8 buffer at concentrations between 0.25 and 1 g/L and irradiated as described above. Please note that the copolymers containing higher amounts of SPA were investigated at lower concentrations due to their higher absorption. To ensure a constant temperature during these measurements, the cuvette was maintained at 15 °C using a thermostat. Upon irradiation with 340 nm, the absorption at 550 nm increases and reaches a maximum after 30 min (Figure 4a). Upon subsequent irradiation at 540 nm, this behavior reverses, and this switching can be cycled at least three times, although the final intensity and switching kinetics decrease after every cycle, likely due to possible oxidation reactions [68] or decomposition of the merocyanin form [70]. Increasing amounts of SPA in the copolymer also increase the absorption and, therefore, the photo-response of the copolymer.

After three consecutive switching cycles, all copolymers display decreased and slower responses to irradiation. To investigate whether the proposed side reactions have any effect on the copolymer structure, both SEC and ^1^H NMR measurements were performed before and after several switching cycles. SEC shows comparable *M*_n_ and dispersities after the switching cycles; however, the corresponding ^1^H NMR spectra differ slightly in the aromatic region. This indicates that the copolymer backbone remains intact, while the SPA moieties are partially affected by the irradiation (Figure S10). Nevertheless, the integrals of the NIPAAm signal at 4 ppm and the SPA signal at 8 ppm still show the same ratio. Hence, we cannot exclude partial degradation, but estimate the overall extent to be within the error of the NMR measurement.

### 3.5. Temperature Response in Aqueous Solution

Responses to both temperature and light can be followed simultaneously by UV–Vis experiments: the photo-response can be followed using the intensity of the absorption band at 550 nm, while heating above the cloud point temperature leads to precipitation and an increase in absorption at 700 nm. An overall decrease in light intensity by a sample can occur for two reasons: (i) at 550 nm, the light intensity decreases due to elastic scattering and the response is measured in absorbance; and (ii) at 700 nm, the light intensity decreases due to inelastic scattering, and the response is given in % transmission. To indicate a separate treatment of these two effects, different ordinates—absorption for light response and % transmission for temperature response—were chosen.

As discussed above, the cloud point for the copolymer is broadened compared to the homopolymer, which is due to different lengths of the PNIPAAm segments. Upon heating, the segments precipitate and scatter the light inelastically, leading to a reduction in the transmission at 700 nm to approximately 10%, independent of the solution concentration. As expected, the effect of the SPA state is larger for the short PNIPAAm segments than for long segments, which precipitate at lower temperature. Hence, at high copolymer concentrations in the setup used, only the precipitation of the long PNIPAAm segments is observed, and the switching state of the SPA has almost no influence on the cloud point temperature. Lowering the concentration of the copolymer decreases the intensity of the signal, but does not increase the effect of the switching of the SPA moiety on the cloud point. A good balance of these two effects was found at a concentration of 0.5 g/L.

The irradiation with either UV (340 nm) or green light (540 nm) was performed under comparable conditions to those described previously. During irradiation at 540 nm, the sample was stirred and heated from 10 to 40 °C in 1 °C steps with an equilibration time of 10 min at each temperature. Afterwards, the cuvette was sealed and cooled in a refrigerator overnight to completely redissolve the copolymer before the measurements were repeated during irradiation at 340 nm. As the copolymer shows no absorbance at 700 nm, the transmittance at 700 nm is then plotted against the temperature for both irradiation periods, and this is exemplarily shown for P(NIPAAm_617_-*co*-SPA_7_) in Figure 4d. The remaining plots are provided in the Supporting Information (Figure S11). For all copolymers, the transmittance at 700 nm decreases drastically upon heating above the cloud point temperature. However, in many cases, the transmittance increases again afterwards, which we attribute to precipitation occurring and partial attachment of some agglomerates to the cuvette wall. As the transmittance increases at the end of the measurement due to agglomeration, the precipitation temperature was determined at the temperature where the minimum transmittance is halved.

However, the effect of salts on the cloud point temperature of PNIPAAm in water is rather complex, and would require further studies to be better understood in the system described here [7,73,74]. P(NIPAAm_617_-*co*-SPA_7_) was measured in deionized water (Figure 4d, green) and in a 50 mM NaCl solution (Figure 4d, yellow curve). Deionized water presents a slightly acidic pH, which leads to the protonation of the SPA moiety. The protonated form of the SPA moiety increases the water solubility of the copolymer because of a constant charge, and effects the light response behavior as seen previously. The constant charge of the copolymer may also shift the cloud point of the system out of the measuring range. As shown by Schild and Tirrell [7], the effect of salt on the LCST behavior is not only dependent on the ionic strength, but rather complex. The ionic strength of the pH 8 TRIS buffer solution is in the same range as that of the 50 mM NaCl solution. This explains the shift in cloud point temperature for P(NIPAAm_617_-*co*-SPA_7_) in deionized water with 50 mM NaCl compared to the pH 8 TRIS buffer solution. These results indicate that the cloud point temperatures may differ in another buffer system at the same pH.

**Figure 5:**
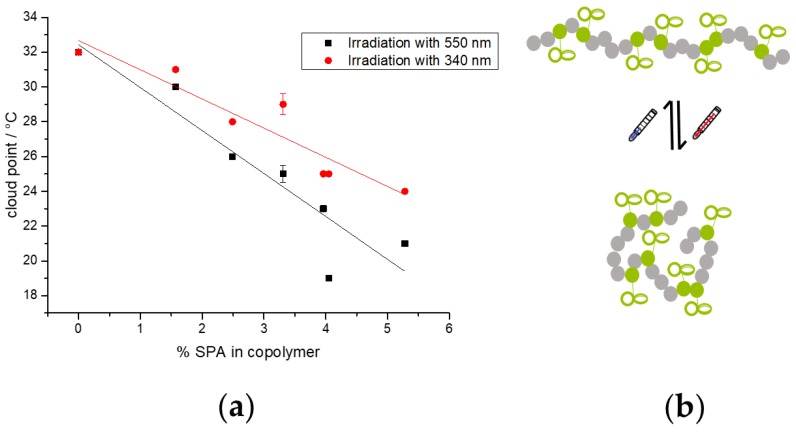
(**a**) Determined transition temperatures between the soluble SP and insoluble MC forms for copolymers containing different amounts of SPA; the red and black lines are just a guide to the eye; (**b**) Schematic view on the transition of the copolymer between soluble and insoluble upon temperature changes.

A clear difference between the cloud point temperatures during irradiation at either 340 or 540 nm can be observed with the difference depending on the SPA content of the copolymers. The values range from 1 °C (P(NIPAAm_311_-*co*-SPA_1_)) to a maximum of 6 °C (P(NIPAAm_617_-*co*-SPA_7_)). The determined differences in cloud point temperature are shown in Figure 5. In general, increasing incorporation of SPA in the copolymer leads to lower cloud points, even below room temperature for high amounts of SPA; as mentioned above, copolymers containing more than 6% SPA are not water soluble at RT. A linear trend is observed for both irradiation states, whereas the copolymers after irradiation at 340 nm feature higher cloud point temperatures.

## 4. Conclusions

We investigated the light and temperature response behavior of moderately disperse P(NIPAAm-*co*-SPA) copolymers of varying molecular weight and composition. Thereby, we found that the temperature response of PNIPAAm and the reversible transition from the spiropyran to merocyanine form of SPA can be combined. The copolymers were synthesized using NMP and characterized using different techniques, including *via* SLS to yield molar masses from 40,000 to 100,000 g/mol and a SPA content as determined *via* ^1^H NMR and UV–Vis spectroscopy between 0% and 5.3%. In the solid state, the copolymers display reversible response to irradiation for a minimum of at least three cycles. Upon transfer to aqueous solution, the irradiation response is more complex but nonetheless comparable to single molecule experiments, and we found the optimal photo-switching conditions to be at 15 °C in pH 8 TRIS buffer. To determine the combined response towards temperature and irradiation, aqueous copolymer solutions were heated during irradiation and the absorbance at 700 nm was taken as an indicator. We observed that a general decrease in the cloud point occurs with increasing SPA content and also the difference between the spiropyran and merocyanine forms is directly reflected in changes in the cloud point temperature.

## Figures and Tables

**Table 1:** Summary of characterization data for the synthesized P(NIPAAm-*co*-SPA) copolymers resulting from varying amounts of SPA in the reaction mixtures.

Composition ^b,d^	x_SPA_ Reaction mixture (%)	*M*_n_^a^	*Ð* ^a^	% SPA ^b^	% SPA ^c^	*M*_w_^d^	Extinction coefficient ^c^/mol·mL^−1^·cm^−1^
PNIPAAm_418_	0	22,900	2.47	0	-	47,300	10,000
P(NIPAAm_311_-*co*-SPA_1_)	1	25,700	1.86	1.5	1.5	35,800	13,000
P(NIPAAm_282_-*co*-SPA_2_)	2	25,800	1.76	2.5	3.6	32,700	29,000
P(NIPAAm_383_-*co*-SPA_4_)	3	25,500	1.58	3.5	5.7	44,800	63,000
P(NIPAAm_538_-*co*-SPA_6_)	4	26,300	1.55	4	7.5	63,400	116,000
P(NIPAAm_617_-*co*-SPA_7_)	5	19,300	1.47	4	10.3	72,700	253,000
P(NIPAAm_1108_-*co*-SPA_17_)	6	22,600	1.41	5.5	10.8	132,300	350,000

^a^ DMAc-SEC, PS calibration; ^b 1^H NMR, 300 MHz, DMSO-d_6_; ^c^ UV–Vis, THF, rounded up to 1000; ^d^ SLS, THF.

## Supplementary Materials

The supplementary materials are available online at http://www.mdpi.com/2073-4360/10/6/645/s1.
