# Supplementary material for: Dual Stimuli-Responsive P(NIPAAm-co-SPA) Copolymers: Synthesis and Response in Solution and in Films
Source: Polymers (Basel). 2018 Jun 9;10(6):645. doi: 10.3390/polym10060645 (PMC6403943; doi:10.3390/polym10060645)
Supplement: Supplementary file 1 [file polymers-10-00645-s001.pdf]

Supporting Information

For

Dual Stimuli-Responsive P(NIPAAm-*co*-SPA) Copolymers: Synthesis and Response in Solution and in Films

Oliver Grimm [1] and Felix H. Schacher [1,2,*]

[1] Institute of Organic Chemistry and Macromolecular Chemistry (IOMC), Friedrich-Schiller-University Jena, Humboldtstraße 10, D-07743 Jena, Germany;
[2] Jena Center for Soft Matter (JCSM), Friedrich-Schiller-University Jena, Philosophenweg 7, D-07743 Jena, Germany; felix.schacher@uni-jena.de
* Correspondence: felix.schacher@uni-jena.de; Tel.: +49-3641 / 9-48250

Synthesis of the copolymers

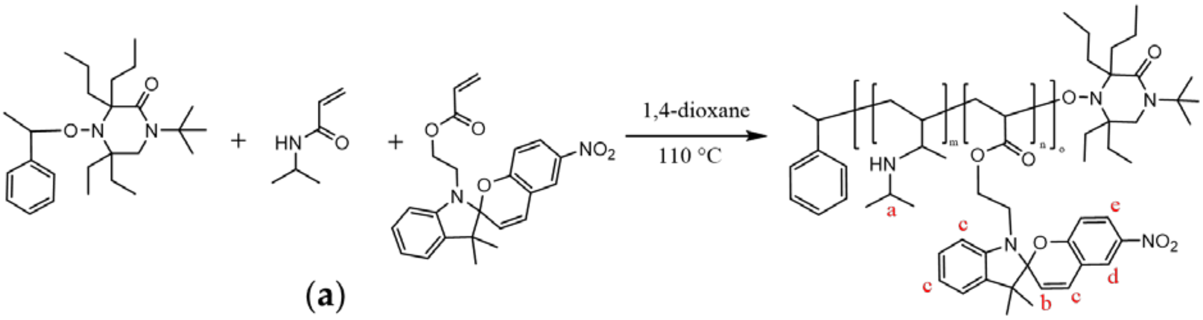

(a)

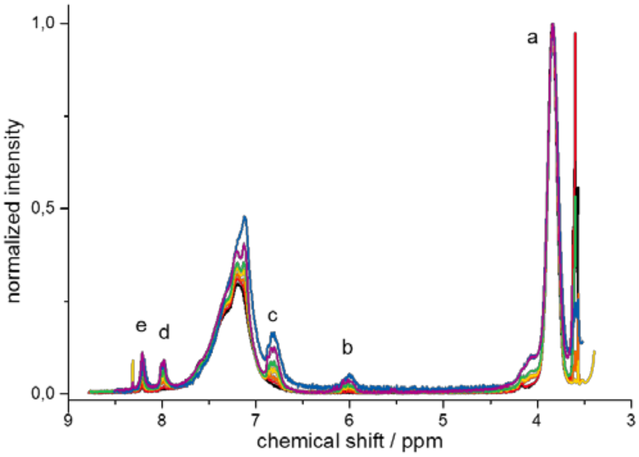

(b)

Composition [b,d]	SPA (%)	SPA (%) [b]
PNIPAAm$_{418}$	0	0
P(NIPAAm$_{311}$-*co*-SPA$_{1}$)	1	1,57
P(NIPAAm$_{282}$-*co*-SPA$_{2}$)	2	2,49
P(NIPAAm$_{383}$-*co*-SPA$_{4}$)	3	3,31
P(NIPAAm$_{538}$-*co*-SPA$_{6}$)	4	3,96
P(NIPAAm$_{617}$-*co*-SPA$_{7}$)	5	4,05
P(NIPAAm$_{1108}$-*co*-SPA$_{17}$)	6	5,28

(c)

Figure S1. a) Reaction scheme depicting the synthesis of the P(NIPAAm-*co*-SPA) copolymers. b) ^{1}H-NMR in DMSO-d$_6$ of the prepared copolymers. The intensity is normalized to the isopropyl-proton of NIPAAm ("a"). c) Composition of the synthesized copolymers calculated from static light scattering and ^{1}H-NMR.

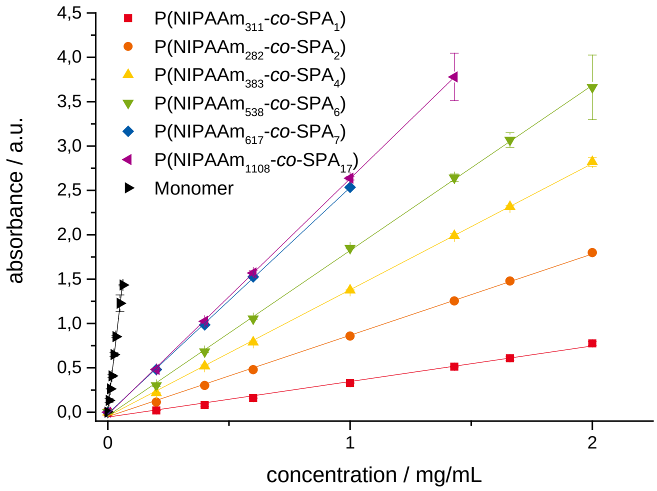

Figure S2. Absorbance of the copolymers and the monomer (SPA) at different concentrations at 330 nm in THF for the determination of the extinction coefficient.

Photo-response in the solid state

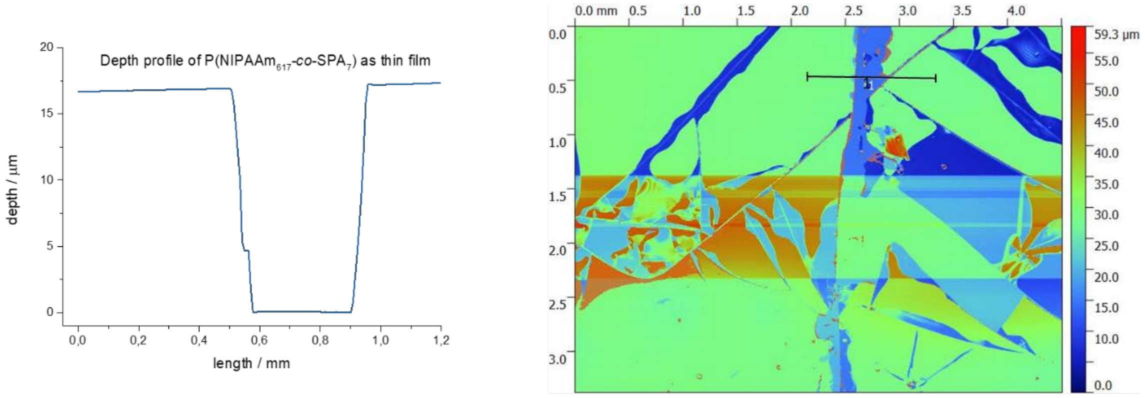

(a) (b)

Figure S3. Analysis of the copolymer films on glass *via* profilometric measurements; a) graph of the thickness, b) investigated area, black bar shows the region of the measured distance.

Table S1. Summarized thickness for the doctor-bladed thin polymer in the obtained films.

Polymer	Thickness / µm
P(NIPAAm$_{311}$-*co*-SPA$_1$)	16
P(NIPAAm$_{282}$-*co*-SPA$_2$)	11
P(NIPAAm$_{383}$-*co*-SPA$_4$)	16
P(NIPAAm$_{538}$-*co*-SPA$_6$)	14
P(NIPAAm$_{617}$-*co*-SPA$_7$)	17
P(NIPAAm$_{1108}$-*co*-SPA$_{17}$)	11

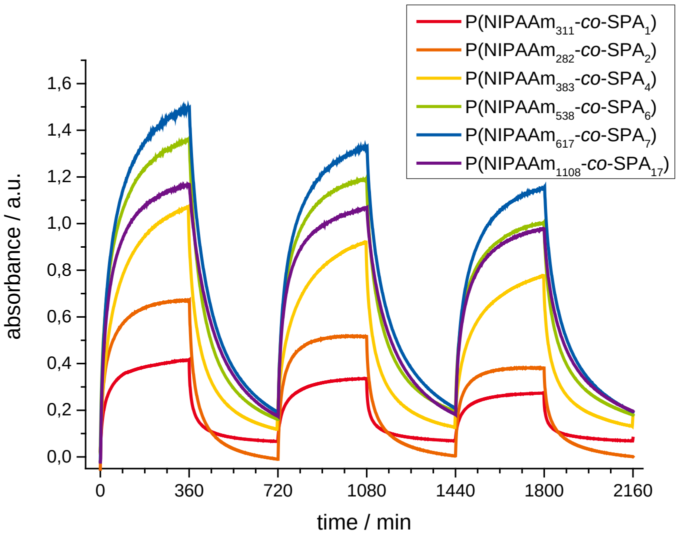

Figure S4. Absorption at 550 nm for different copolymer films on microscope slides while irradiating at 365 or 590 nm for 6 h each.

Photo-response in aqueous solution

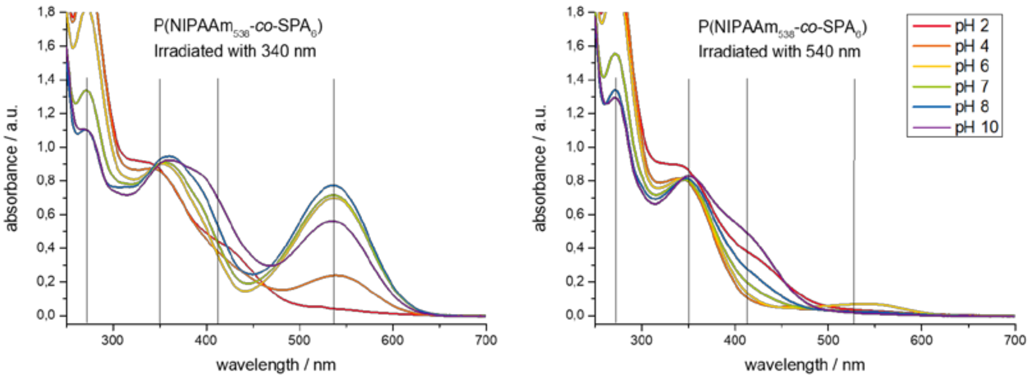

(a) (b)

Figure S5. Absorption spectra of P(NIPAAm$_{538}$-*co*-SPA$_6$) at different pH values after irradiating at 340 (a) or 540 nm (b) for 30 min at 15 °C.

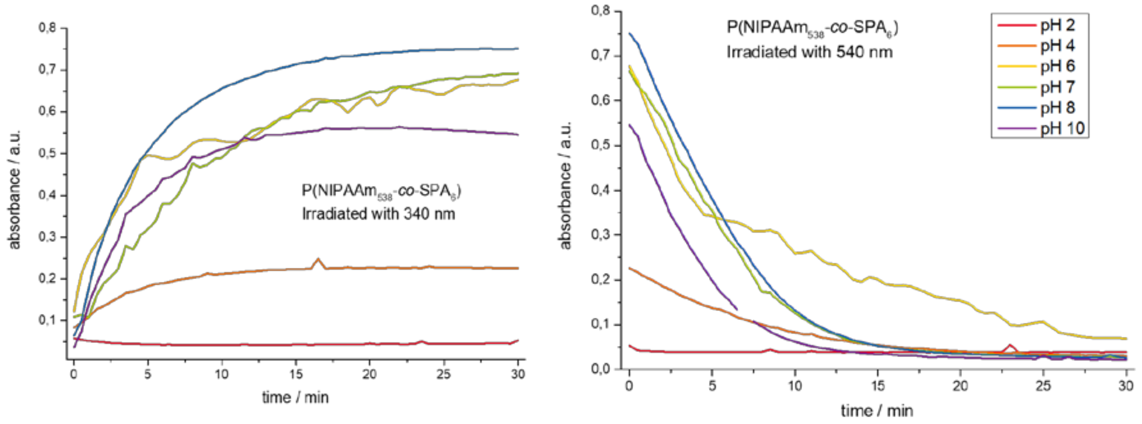

(a) (b)

Figure S6. Change of absorption at 550 nm of P(NIPAAm$_{538}$-*co*-SPA$_6$) at different pH values during irradiation with 340 nm (a) and 540 nm (b) light at 15 °C.

Kinetics of the photo-response

The following formula was used to calculate the reaction kinetics:

$$[y] = [y_0] + Ae^{-kt}$$

All irradiation traces were fitted using this formula, and the required parameters plotted in the following graphs.

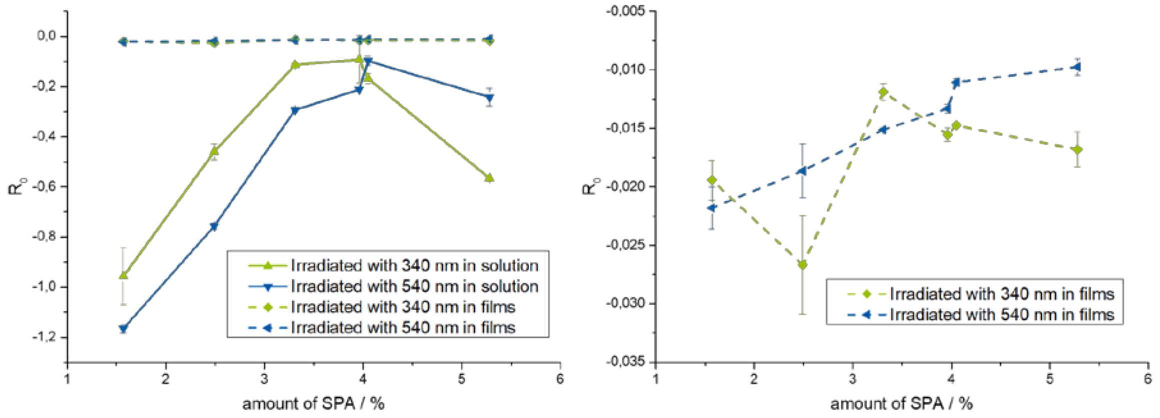

(a) (b)

Figure S7. Reaction constant of the synthesized SPA containing copolymers in solution and in films (a), and a detailed view of the reaction constant for the films (b).

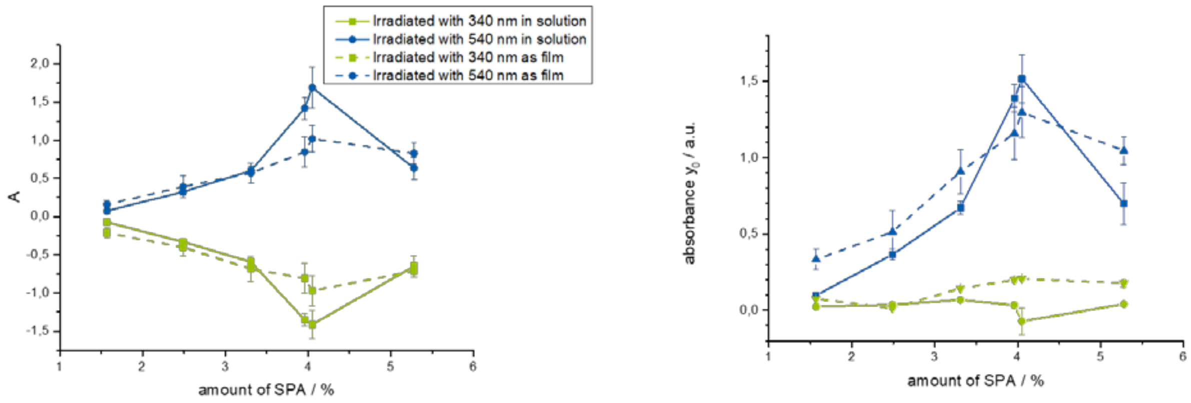

(a) (b)

Figure S8. Fitting parameters A (a) and y_0 (b) used for the kinetic description of the photo-response of the copolymers in solution (straight line) and in films (dotted line).

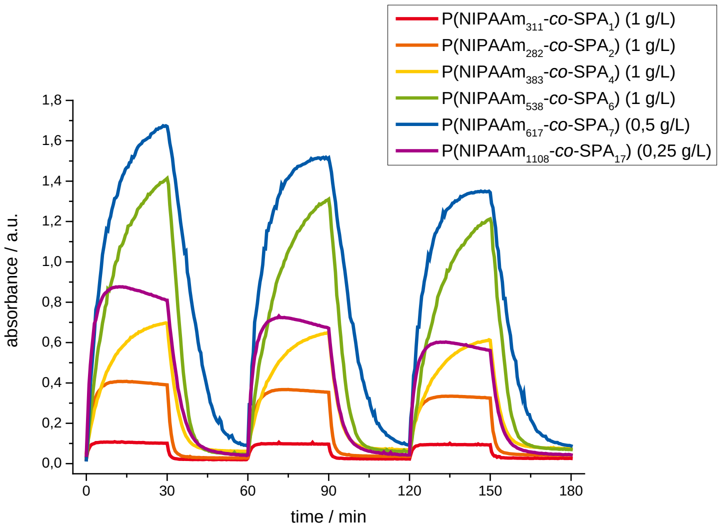

Figure S9. Absorbance of the copolymers containing SPA moieties after irradiation with a 200 W Hg(Xe) lamp. Three cycles were performed by alternating irradiation with a 340 nm filter and a 540 nm filter for 30 min. The concentration of the copolymers in pH 8 TRIS buffer was lowered for copolymers containing higher amounts of SPA.

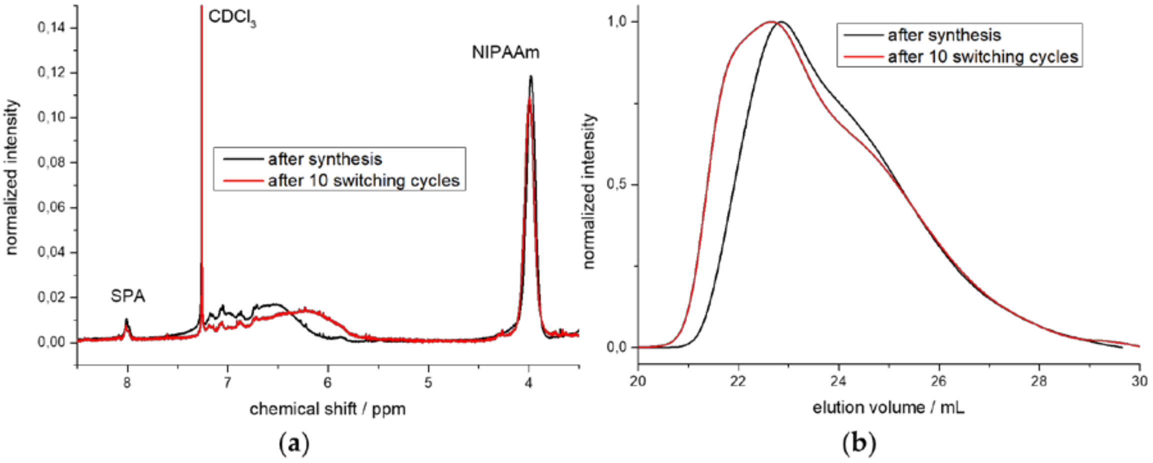

Figure S10. a: ^{1}H-NMR of P(NIPAAm$_{383}$-*co*-SPA$_4$) in $CDCl_3$ before and after various switching cycles in a pH 8 buffer solution as used for further experiments. b: SEC elution traces of the same copolymer.

Temperature response in aqueous solution

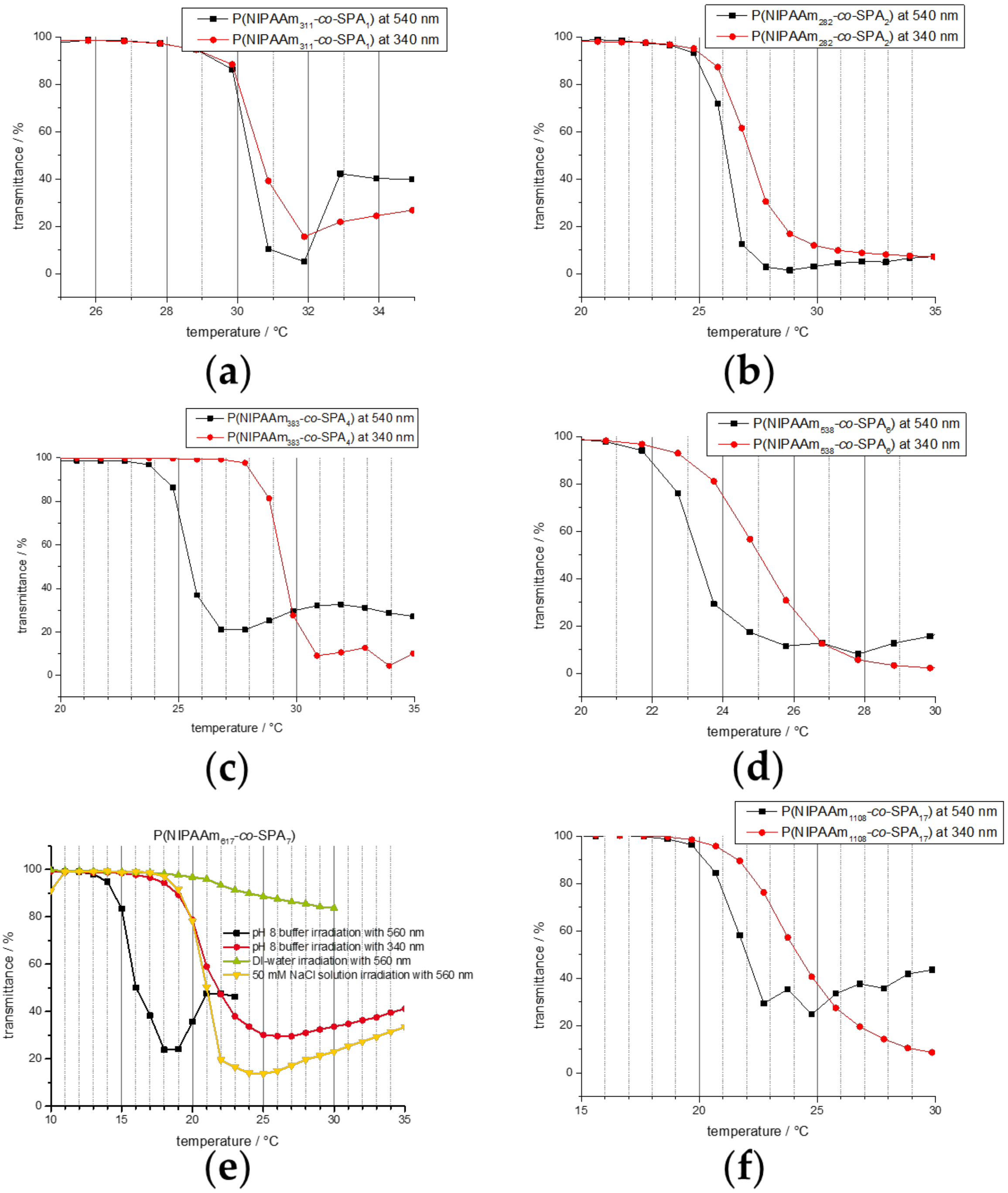

Figure S11. Transmittance at 700 nm of copolymers dissolved in a pH 8 TRIS buffer at different temperatures while irradiating with a 200 W Hg(Xe)-lamp and different wavelength filters. The copolymer P(NIPAAm$_{617}$-*co*-SPA$_7$) (e) was additionally measured in deionized water (green) and NaCl-containing water (yellow) while irradiated at 540 nm.

Table S2. Precipitation temperatures of the different copolymers while irradiating with UV (340 nm) or green light (540 nm). The precipitation temperature was determined as the temperature where the transmission was halved from Figure S11.

Polymer	Transition Temperature under UV Light / °C	Transition Temperature under green Light / °C
P(NIPAAm$_{311}$-*co*-SPA$_{1}$)	30	31
P(NIPAAm$_{282}$-*co*-SPA$_{2}$)	26	28
P(NIPAAm$_{383}$-*co*-SPA$_{4}$)	25	29
P(NIPAAm$_{538}$-*co*-SPA$_{6}$)	23	25
P(NIPAAm$_{617}$-*co*-SPA$_{7}$)	19	25
P(NIPAAm$_{1108}$-*co*-SPA$_{17}$)	21	24
